# A Distinguished Physician-Pharmacist—His Great Discovery, Ether-Anesthesia*Read at the Forty-fifth Annual Meeting of the American Pharmaceutical Association, held at Lake Minnetonka, Minn., August, 1897.

**Published:** 1897-11

**Authors:** Joseph Jacobs

**Affiliations:** Atlanta, Ga.


### A DISTINGUISHED PHYSICIAN-PHARMACIST—HIS GREAT DISCOVERY, ETHER-ANÆSTHESIA.*

BY JOSEPH JACOBS.

In our practical age, that is so prolific of discoveries and improvements in science, and in which the spirit of mercantilism tends to absorb and dominate all thought and action, it is not inappropriate for us, at times, to turn away from our discussions of technical topics and revert to subjects of historical and sentimental interest. Our country, so vast in area and so varied in the composite elements that constitute its social framework, in point of age and historical moment, is still an adolescent in the family of nations.

But in the vigor of its youth and rapidity of its growth, the memorial materials of its history are being overlooked. Too often are they swept from the pathway of progress or neglected, and in future years it will be regretted that our generation has omitted to preserve the true records of American achievements.

I have thought it therefore, not ill-timed that I should pre-

*Read at the Forty-fifth Annual Meeting of the American Pharmaceutical Association, held at Lake Minnetonka, Minn., August, 1897.

sent the facts to this Association connected with the discovery by an American physician-pharmacist of one of the greatest boons ever conferred upon humanity in the history of the world.

As the man who made the discovery for many years owned and conducted a pharmacy, and thus connected his life and name with our honored profession, it is entirely proper that we should share and feel pride in the lofty honor that history will accord to his memory.

I refer to the discovery of the uses of ether as an anæsthetic in surgical operations, and to Crawford W. Long, of Georgia, the physician-pharmacist who first employed this anæsthetic in those operations. True, he was a practicing physician, but for many years he devoted himself to the interests of pharmacy, and was intimate with the pharmacists of his day in social and business relations, and it is entirely proper that we, as pharmacists, shall treasure the glory of his greatness as, in part, a portion of our own history.

There have been four claimants for the great honor of this discovery, all Americans: First, Crawford W. Long, Physician-Pharmacist, of Georgia; second, Charles T. Jackson, Physician-Scientist, of Massachusetts; third, Wm. T. G. Morton, Dentist, of Massachusetts; fourth, Horace Wells, Dentist, of Connecticut.

For fifty years the medical journals and scientific publications have contained conflicting articles upon the subject of the discovery of the use of ether; and the controversy does not seem ended yet, for on the 30th of September, 1896, there was a public celebration at Boston, Mass., at the Massachusetts General Hospital, of the "Fiftieth Anniversary of the Discovery of Anæsthesia by Wm. T. G. Morton," which celebration gained wide notice in newspaper publications.

I do not hope that what I here say will be uncontroverted, and I frankly confess that I may be animated by personal feelings in venturing to advocate the claims of Crawford W. Long before this intelligent and representative body; for I knew him well, and in my youth learned to reverence and admire him while an employee and pupil in his pharmacy, and shall ever remain grateful for his kind friendship and valued instruction; but, as the friends of the other claimants continue to put forth and revive their claims, to which the attention of all the members of this body have doubtless been directed, it is but fair and just, while the process of moulding the final verdict is going on, that the distinguished Georgian shall have the merits of his title fully disclosed.

Each of the claimants thus far has had the recognition of organized bodies of men.

The claims of Chas. T. Jackson were recognized by scientific bodies in France and Prussia, before whom the claims of none of the others were presented.

The claims of Horace Wells have been recognized by the State of Connecticut, that State having erected a monument at Hartford inscribed, "Horace Wells, who discovered Anæsthesia Nov. 2, 1844."

A citizen of Boston, Thos. T. Lee, in honor of Jackson or Morton (it is not decided), erected a monument to the "Unknown Discoverer of Anæsthesia." Its main inscription is as follows: "To commemorate the discovery that inhaling of ether causes insensibility to pain, first proven to the world at the Massachusetts General Hospital in Boston, MDCCCXLVI."

The claims of Dr. Long have been recognized by the Georgia State Medical Association, by the American Association of Eclectic Physicians, and by the State of Georgia in hanging his portrait on the walls of her capitol among those of the great men of the State and country.

I shall endeavor, in as succinct a manner as possible, and in a fair manner to all, to present the facts of the controversy. As to the time and main circumstances of the first use of anæsthetics by the four claimants, the following is a fair statement:

Crawford W. Long, at Jefferson, Jackson County, Georgia, entirpated a tumor from the neck of James M. Venable, while he was under the influence of ether, without pain to the patient, on the 30th day of March, 1842.

Horace Wells subjected himself to the effect of nitrous oxide gas, and had one of his own teeth extracted without pain, to test the value of the gas as an anæsthetic, on Dec. 11, 1844.

Chas. T. Jackson did not administer ether in any operation, but it is claimed suggested its use to Dr. W. T. G. Morton, Sept. 30th, 1846.

W. T. G. Morton gave ether to a Mr. Frost, Sept. 30th, 1846, and extracted a tooth without pain.

The dates and the persons on whom ether was used in the four cases stated, are unquestionably established. In the case of Dr. Long, the patient's affidavit, and those of four students who were in Dr. Long's office, sustain Dr. Long's written statement. And in each of the three other cases the times at which the anæsthetics were used are as amply verified and fixed.

Hence, it is beyond dispute that Dr. Long's use of ether as an anæsthetic in surgery antedates Wells' use of nitrous oxide gas two years and eight months, and the use by Morton of ether by four years and six months.

If this be true, it will be asked, why has not Dr. Long been finally and fully recognized by mankind as the true and real first discoverer of the use of ether as a preventive of pain in surgical operations? This question can be answered, in part, by suggesting that the subject has been clouded in doubt, not as to the dates of the use of ether, but because of the controversy that grew up over the rival claims of Wells and Morton and Jackson before the United States Congress, and in the persistency with which the friends of these claimants have urged and repeated their claims.

At the suggestion of Dr. Charles T. Jackson, as it is claimed by the advocates of Jackson (and this is fully substantiated), Dr. W. T. G. Morton, who was his partner in business, went before the surgeons of the Massachusetts Hospital of Surgery in the fall of 1846, four years after Dr. Long's discovery, and suggested that they test the efficacy of a new agent for preventing pain in surgery which he called "Letheon," and for which he and Dr. Jackson had applied for a patent from the United States Government. Drs. Warren, Haywood and Bigelow, surgeons in charge of the hospital, consented to try the "Letheon," which was nothing but ether disguised by aromatic oils. They, on the 16th of October, 1846, used it in removing a tumor from a young man, and afterwards, on November 7th, performed the operations of amputating above the knee and in the excision of the lower jaw—all successfully and without pain to the patients. On the 27th of October, the following affidavit was made and taken, to wit: "On this 27th day of October, 1846, personally came before me, Charles T. Jackson and Wm. T. G. Morton, and made oath *that they do verily believe themselves to be the original and first inventors of the improvement* hereinbefore described (alluding to ether as an anæsthetic), and they do not know or believe the same to have ever before been known or used, and they are citizens of the U. S. A.—(Signed), R. J. Eddy, Justice of the Peace."

Jackson and Morton applied for a patent in their joint name, but Jackson, fearing the censure of the Massachusetts Medical Society on the score of ethics, insisted on assigning to Morton all his rights under the patent, and that the patent issue in Morton's name, but took a private writing that he was to get ten per cent. of all made out of it.

As soon as the surgeons of the Massachusetts General Hospital were confirmed in the belief of the success of the discovery, Dr. H. J. Bigelow, one of the hospital surgeons, wrote an account of the use of "Letheon" (to him then an unknown or secret substance), which was published in the *Medical Examiner* for December, 1846.

Morton commenced to sell his patent rights, and succeeded in disposing of a number of privileges to dentists and others for various territories.

In 1847, Drs. Jackson and Morton fell into differences, and waged a war of pamphlets, involving their respective claims to the discovery, and when in 1854, Morton presented a memorial to Congress asking that the Government pay him a large sum of money for the use of ether, and in honor of him being its discoverer as an anæsthetic, the friends of Wells and Jackson, as well as those of Dr. Long, interposed their claims and defeated the movement.

The claims of Wells were by Congress, and now are generally, conceded to extend no further than to the use of nitrous oxide gas, which for the purposes of general surgery can not be substituted for ether.

The proofs are numerous in the form of affidavits of mutual associates of Morton and Jackson that Jackson did not in any way practically use or demonstrate the use of ether, and that Morton, who did use it, used it upon the suggestion of Jackson. The only claimant who originally conceived the use of ether in surgery, and himself experimented with its use to the extent of practically demonstrating its efficacy, was Dr. Long, and this was accomplished by him more than four years prior to the time of its use by Morton. Dr. Long did not publish in any printed form his discovery to the world until 1849, and then in the *Southern Medical and Surgical Journal*, but he made the discovery known to the students in his office and to practicing physicians at Athens, Georgia, and in all the territory surrounding him.

He made no secret of his discovery, but talked about it on every appropriate occasion to medical men, and was waiting for the opportunity to test it in a capital operation before writing about it in the scientific journals of the day. Meanwhile those operations before described were accomplished at the Massachusetts General Hospital, and through Dr. Bigelow the uses of ether were published to the world.

An examination of the evidence will clearly show that Dr. Jackson never at any time practically applied ether in a surgical operation, but merely suggested its use to Dr. Morton; that Dr. Morton did, at the suggestion of Dr. Jackson, apply and use ether successfully, but that his intention was to keep the process a secret, shown by his taking a patent on it and in all his conduct; for, when, at the instance of Dr. Jackson he permitted its use at the Massachusetts General Hospital, it was introduced and described as "Letheon," a secret compound. By reason of differences between him and Dr. Jackson, copartners in the patent, the nature of the substance became known to the hospital surgeons, and *they* published it to the world.

When the controversy between Morton and Jackson and Wells was raging before Congress, Dr. Jackson learned that the use of ether had been known to Dr. Crawford W. Long, in March, 1842. and in order to defeat the claims of Morton he made a lengthy journey to Athens, Georgia, to see Dr. Long, and tried to induce him to unite with him in jointly claiming the discovery. This Dr. Long refused, simply stating that he stood upon the facts. Dr. Long made no effort before Congress to obtain an appropriation, but the facts of his discovery were presented by Senator Wm. C. Dawson, of Georgia, and these facts went far to defeat the claims of Morton, Wells and Jackson to a money donation from the General Government. Dr. Long always said that the only reward he wished was to be considered a "benefactor of his race."

As between Jackson and Morton, it has been shown in an article prepared by Lord & Lord, attorneys for Dr. Jackson, and published in *Littell's Living Age* during the time of the controversy between them, by more than a score of affidavits from men associated with Drs. Jackson and Morton in 1846, that Morton never claimed to have had the original idea of using ether, that he invariably attributed the suggestion of its use to Dr. Jackson. He, Morton, was the mere agent, an automaton in the hands of Dr. Jackson. These affidavits, made in 1846–1847, while the discussion was at its height, almost unanimously state that Dr. Morton had acknowledged this fact in the presence of the affiants, and that he clearly and repeatedly stated that what he knew about ether as an anæsthetic was derived from the suggestion and teachings of Dr. Jackson.

I quote from only one of this score of affidavits: it was made by H. J. Payne, a Surgeon-Dentist of Troy, New York, 12th April, 1848:

"On the 2nd day of Jan., 1847, I went to Boston and sought an interview with Dr. Morton. I had a protracted interview with him with respect to the use and effect of the vapor of ether, its discovery, and the patent that had been taken thereupon. During this interview Dr. Morton stated emphatically and repeatedly that Dr. Chas. T. Jackson, of Boston, was the sole discoverer of this new agent for producing insensibility to pain, and that Dr. Jackson had communicated it to him. Furthermore, that all the knowledge he possessed in relation to its properties and application had come to him from Dr. Jackson, and that he never had any idea of applying sulphuric ether, or that sulphuric ether could be applied for the aforesaid purposes, until Dr. Jackson had suggested it to him, and had given him full instructions. I then questioned Dr. Morton with regard to the patent, how he came to have an interest in it, etc. He replied that he had been very fortunate in effecting an arrangement with Dr. Jackson before any one else had the opportunity, and that he was the first man to whom the discovery had been communicated by Dr. Jackson, and added, 'I have made a great bargain.'"

Now there are many such affidavits on record, showing that while Dr. Morton may have used ether in 1846, he never at any time conceived such use as an original proposition, but derived all his knowledge of its properties and the suggestion of such use from Jackson.

Here then the controversy narrows down to Jackson and Long, but it must be determined as to priority in favor of Dr. Long; for when Jackson was hard pressed by Morton during the effort before Congress, he turned to Dr. Long, who had published an account of his use of ether in 1842 in the *Southern Surgical and Medical Journal* of 1849, and when Dr. Jackson had seen Dr. Long at Athens, Georgia, and had carefully studied the evidences of Dr. Long's use of ether in 1842, and of his having made it known to his community and to professional men with whom he was associated, he returned to his Boston home, himself convinced that Dr. Long had of his own original intuitions thought out the utility of ether, and had successfully applied it as a preventive of pain in surgical operations. And Jackson himself has admitted Dr. Long's claim to be the true and real discoverer in no less solemn manner than a written communication to a medical journal over his own signature. In the *Boston Medical and Surgical Journal* of April 11, 1861, Dr. Chas. T. Jackson says that he visited Dr. Long at Athens, Ga., on March 8, 1854, to examine into his claims to being the first to use sulphuric ether as an anæsthetic in surgery, and continuing says:

"From the documents shown me by Dr. Long, it appears that he used sulphuric ether as an anæsthetic—

"First.—On March 30th, 1842, when he extirpated a small glandular tumor from the neck of James M. Venable, in Jefferson, Ga. (now dead).

"Second.—On the 3d of July, 1842, in the amputation of the toe of a negro boy belonging to Mrs. Hemphill, of Jackson county, Ga.

"Third.—On September 9th, 1843, in the extirpation of a tumor from the head of Mary Vincent, of Jackson county, Ga.

"Fourth.—On January 8th, 1845, in the amputation of the finger of a negro boy belonging to Ralph Bailey, of Jackson county, Ga.

"Copies of letters and depositions proving these operations with ether, were all shown to me by Dr. Long. He also referred me to physicians who knew of the operations at the time."

Dr. J. Marion Sims, of New York, in 1877, in an article in the *Virginia Medical Monthly*, quotes the above extract from the article of Dr. Chas. T. Jackson, and adds: "The above extract from Dr. Jackson's paper to the *Boston Medical Journal*, recognizes Long's claim to being the first to produce anæsthesia for surgical operations, but it does not tell the whole story of Dr. Jackson's visit to Dr. Long. Dr. Long has furnished me with all the evidence, consisting of affidavits, certificates, book entries, etc., that Dr. Jackson examined. He had also written to me fully on the subject, and every fact that I have stated can be sustained by documentary evidence.

In one of Dr. Long's letters to me (Nov. 5th, 1876), he says: 'Dr. Chas. T. Jackson came to Georgia and spent two days with me at Athens, most of the time in my office; examining dates and certificates, establishing the time, etc., of my operations, he expressed himself as satisfied with the correctness of my claim to the first use of ether as an anæsthetic in surgical operations. Dr. Jackson informed me he would go from Athens to Dahlonega, Ga., and as I knew he must pass through Jefferson, where I resided up to 1850, and where my first operations under ether were performed, I requested him to stop in Jefferson and see some of the physicians there who witnessed or knew of the operations or were familiar with them from common report. Dr. Jackson spent one or more days in Jefferson, and on his return, expressed himself as satisfied with the testimony. In Dr. Jackson's communication to the *Boston Medical and Surgical Journal*, he neglected to say anything of the information he received while in Jefferson, although he admitted to me on his return that the evidence was perfectly satisfactory.'" Dr. Sims, continuing, says: "The Hon. C. W. Andrews, of Madison, Ga., informs me that he was in Dr. Long's employ and in his office when Dr. Jackson spent a whole day with Dr. Long in comparing notes and talking over the subject of etherization, and it seems that the real object of Dr. Jackson's visit to Dr. Long was to induce Dr. Long to unite with him in laying their conjoint claims before Congress as the real discoverers of anæsthesia, as opposed to those of Morton. Jackson was willing to concede to Long the honor of being the first to use ether in surgical operations, but wished Long to concede to him the honor of priority in making discovery of the principle of anæsthesia when he inhaled ether to relieve the pain and difficulty of breathing after inhaling chlorine gas (as Sir Humphrey Davy had done before).

Dr. Long says, February 8th, 1877, "In our conversation I understood Dr. Jackson to yield the point of priority to me, and so did the Hon. C. W. Andrews.

"I did not admit to him that he was the first to make the discovery—leaving to me its practical application—and when he proposed to me to unite our claims—he to claim the discovery, and I to claim its first practical use in surgical operations—I positively refused. I was satisfied I was entitled to the credit of the discovery, as well as of the first practical use of ether in surgical operations."

Dr. Jackson is further quoted by Dr. Sims as having said to Dr. Long during his visit to Athens: "You have the advantage of priority in date and in the first use of ether as an anæsthetic, but we have the advantage of the priority of publication."

But Dr. Sims, continuing, says: "Now, upon this point Dr.

Jackson is evidently mistaken as to his advantage of priority of publication."

For abundant and indisputable evidence is given that Dr. Long did exhibit to medical men and to the community at large his operations under the influence of ether in 1842, while Wells, Morton and Jackson made no exhibit until as late as 1844 and 1846. It is true Dr. Long may not have published his discovery in the medical journals of the country, nor does it appear that the other claimants did; but exhibiting their experiments in the large cities of New York and Boston, of course better facilities were offered for disseminating the facts throughout the medical world. However, abundant evidence has been produced by Dr. Long to prove that he made no secret of his discovery, but, on the contrary, communicated it as rapidly to the medical fraternity as his restricted and limited facilities would permit, and the fact that he did not perhaps publish it through the medical journals makes him none the less the true discoverer.

Whatever credit may be due to the memory of Jackson and Morton and Wells for their researches and their use of anæsthetics, and whatever honor may attach to the eminent surgeons of the Massachusetts General Hospital for publishing the facts at home and abroad, the real glory of the first discovery and proof of the efficacy of ether for the prevention of pain in surgery must be finally awarded to Crawford W. Long, the eminent Georgian and lamented physician-pharmacist.

On my last visit to my old home in Athens, Georgia, I stood at the grave of this good and great man. On the banks of the beautiful Oconee river, in our Southland, with no monument of imposing grandeur, his resting-place marked alone with the simple marble within the power of loved ones to place there, is the grave of the great discoverer; and the flowers that bloom in sweet profusion on the earth above him seemed to betoken the lofty sentiment I have heard him so often express, that he wished no recompense or reward for the priceless boon he had conferred on humanity, save the recognition that he had been a "benefactor of mankind."

*Atlanta, Ga., Aug. 7, 1897.*

